# Supplementary material for: 400nm ultra-broadband gratings for near-single-cycle 100 Petawatt lasers
Source: Nat Commun. 2023 Jun 19;14:3632. doi: 10.1038/s41467-023-39164-3 (PMC10279661; doi:10.1038/s41467-023-39164-3)
Supplement: Supplementary file 1 — Supplementary Information [file 41467_2023_39164_MOESM1_ESM.pdf]

Supplementary Information for:

400 nm ultra-broadband gratings for near-single-cycle 100 Petawatt lasers

Yuxing Han[1,2,3], Zhaoyang Li[4,5,*], Yibin Zhang[1,2,3], Fanyu Kong[1,3], Hongchao Cao[1,3], Yunxia Jin[1,3,6,*], Yuxin Leng[4], Ruxin Li[4,5], Jianda Shao[1,3,6,7]

[1]Laboratory of Thin Film Optics, Shanghai Institute of Optics and Fine Mechanics, Chinese Academy of Sciences, Shanghai 201800, China

[2]Center of Laboratory of Materials Science and Optoelectronics Engineering, University of Chinese Academy of Sciences, Beijing 100049, China

[3]Key Laboratory of Materials for High Power Laser, Chinese Academy of Sciences, Shanghai 201800, China

[4]State Key Laboratory of High Field Laser Physics, Shanghai Institute of Optics and Fine Mechanics, Chinese Academy of Sciences, Shanghai 201800, China

[5]Zhangjiang Laboratory, Shanghai 201210, China

[6]CAS Center for Excellence in Ultra-Intense Laser Science, Chinese Academy of Sciences, Shanghai 201800, China

[7]Hangzhou Institute for Advanced Study, University of Chinese Academy of Sciences, Hangzhou 310024, China.

*lizy@zjlab.ac.cn, yxjin@siom.ac.cn and jdshao@siom.ac.cn

Supplementary Note 1:

Example design of a stretcher-compressor system for a 100 PW laser. Based on the ultra-broadband high-energy amplification by WNOPCPA[1], an example of the stretcher-compressor system is designed using the developed 1443 lines/mm ultra-broadband gold gratings in the main article. The designed stretcher-compressor system can directly support 6 fs–600 J–100 PW lasers. When adding a single-stage thin-film/thin-plate compressor (or named thin-film/thin-plate post-compression) after the grating compressor, ~1.2 fs–600 J–500 PW lasers would be possible[1,2]. Detailed parameters of the stretcher-compressor system are given in Supplementary Table 1.

Supplementary Table 1 Parameters of the stretcher-compressor system and gain materials in the amplifier.

**Compressor**			
Incident angle	69°	Perpendicular length	600 mm (×2)
Azimuthal angle	0°	Spectral range	730-1130 nm
Input energy	~900 J	Beam size	300 mm× 600 mm
Output energy	~600 J	Energy fluence (G4)	~0.12 J cm^{-2}
Grating 1/4 size	870 mm × 600 mm	Grating 2/3 size	1370 × 600 mm
**Stretcher**			
Incident angle	68.8275°	Perpendicular length	-299.45 mm (×4)
Azimuthal angle	0°	Spectral range	730-1130 nm
**Gain materials in the amplifier chain**			
Crystal type	Type-I LBO	Total thickness	54 mm
Angle θ	90°	Angle ϕ	12°
Light type in crystal	Ordinary light		

Supplementary Fig. 1 shows the designed compressor, which meets the following requirements: first, the geometrical layout of the compressor, i.e., the second and the third gratings should not interrupt the input and the output beams; second, the second and the third gratings should support the spectral range of 730–1130 nm without any clipping; third, the designed energy fluence at the last grating (G4) surface is around 0.12 J cm^{-2}, which should be lower than the reported damage threshold of 0.15–0.2 J cm^{-2} for few femtosecond pulses[3,4]. The input beam is shaped with a rectangle aperture of 300 mm× 600 mm, and the short side (300 mm) is along the grating dispersion direction. Supplementary Fig. 1 shows the detailed engineering design of the compressor.

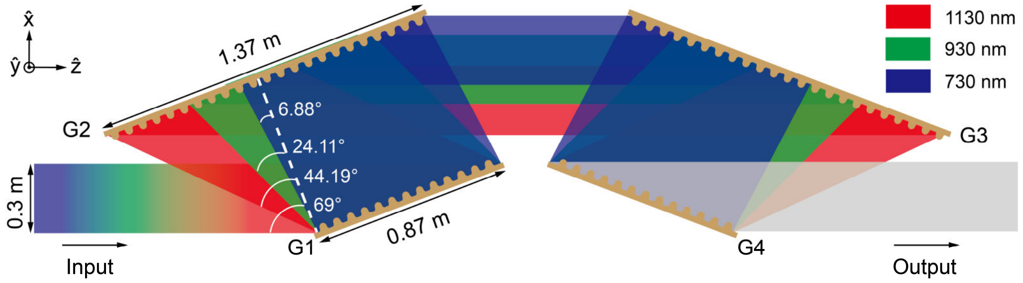

Supplementary Fig. 1 Compressor design. Geometrical parameters of the four-grating single-pass compressor are shown. G1–G4 are 400 nm ultra-broadband gratings.

Because the beam transport system consists of reflection/achromatic optics, only the LBO crystals in the amplifier chain introduce material dispersion. Refer to Supplementary Ref. [1], as given in Tab. S1, the total length of LBO crystals is 54 mm. The signal is the ordinary light (o-light) in LBO, and then using angles θ and ϕ in LBO and signal wavelengths, the wavelength-dependent refractive indices, as well as the spectral phase, can be calculated by the Sellmeier equation.

Supplementary Fig. 2 shows the engineering design of the stretcher, which minimizes the residual spectral phase of the stretcher-amplification-compressor system. The stretcher is also based on the developed 1443 lines/mm ultra-broadband gold gratings and has a four-pass configuration by introducing two roof mirrors. The incident angle and the perpendicular length are slightly changed for the dispersion management. Here, we only give a general optical configuration that one can use in either the Offner-type or the Martinez-type configuration. Reflection concave and convex mirrors should be used for imaging to avoid introducing extra material dispersion.

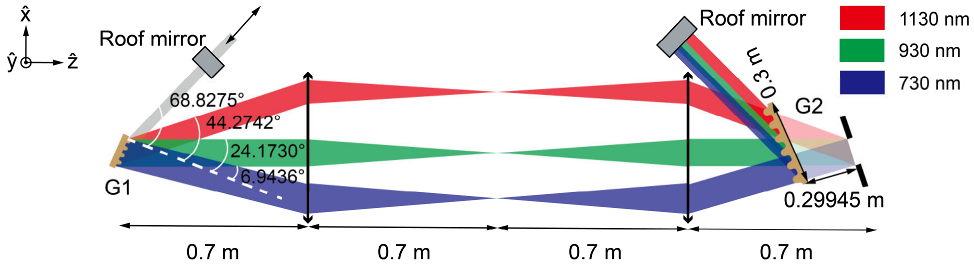

Supplementary Fig. 2 Stretcher design. Geometrical parameters of the two-grating four-pass stretcher are shown. G1–G2 is 400 nm ultra-broadband gratings.

In both the stretcher and the compressor, the incident angle is designed at ~69°. Supplementary Fig. 3 shows that the developed 1443 lines/mm ultra-broadband gold grating can supply > 90% diffraction efficiencies for the 750–1130 nm spectral range. Only at 730–750 nm, the diffraction efficiency decreases to around 75%, which will not obviously affect the pulse compression.

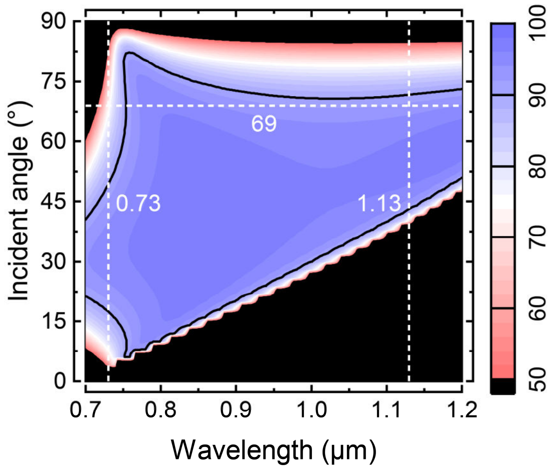

Supplementary Fig. 3 Diffraction efficiency. Gratings in the stretcher-compressor system work at the condition of incident angles ~69° and spectral range 730–1130 nm.

Supplementary Fig. 4(a) shows the group delays introduced by the designed stretcher + amplifier

and the compressor, respectively, and the chirped pulse duration is around 4.5 ns. Figs. S4(a-b) show that the stretching ratio at the short wave (730–860 nm) is smaller than that at the long wave (900–1130 nm). Here, for a 300 mm × 600 mm rectangle beam and an ~900 J amplified pulse energy, the peak intensity is lower than 0.4 GW cm^{-2}, which will not damage the amplification crystals, the transport mirrors, and the compression gratings. Supplementary Fig. 4(c) shows the residual group delay of the stretcher-amplification-compressor system, and the black curve illustrates the corresponding pulse in Supplementary Fig. 4(d), which is around 30% of the Fourier-transform-limit (FTL) pulse (red curve). The shadow in Supplementary Fig. 4(c) is the tunable range of Dazzler, a commercial spectral phase controller[5], which covers the residual group delay, and then by using it, the FTL pulse can be obtained eventually.

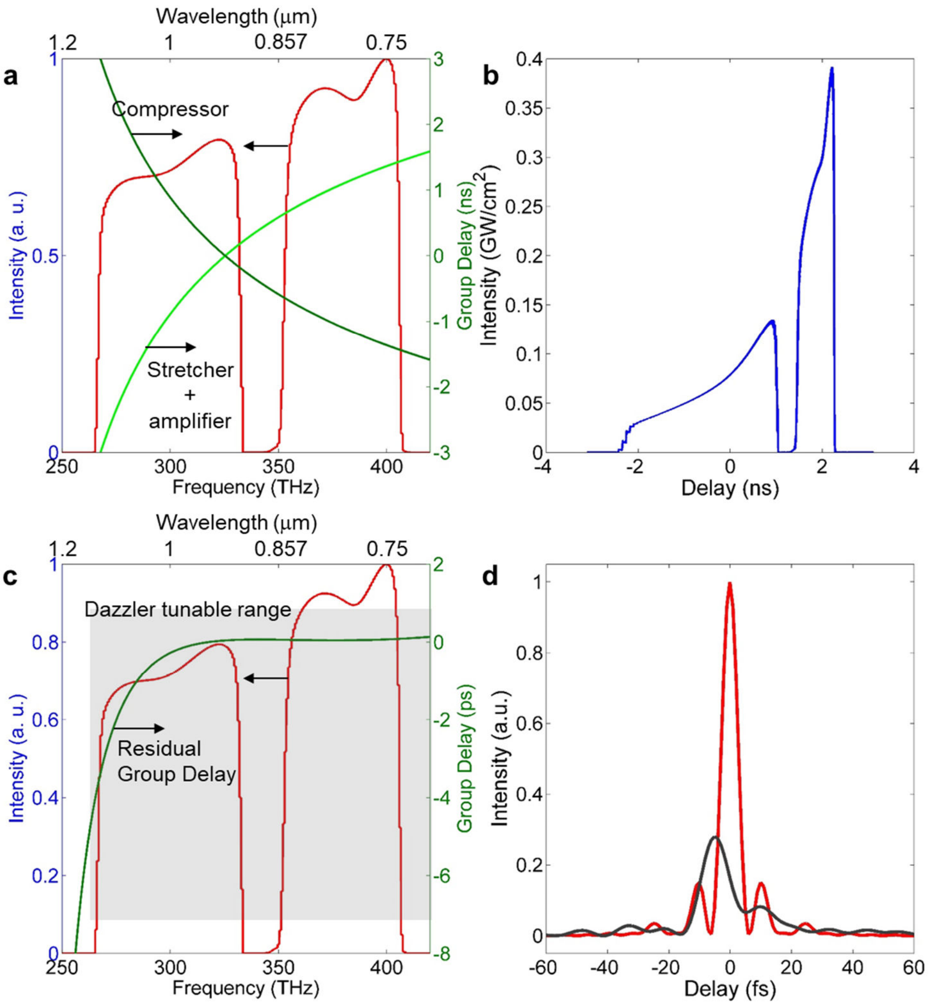

Supplementary Fig. 4 Spectral-dependent group delays, chirped and compressed pulses. (a) Pulse spectrum and group delays of stretcher + amplifier and compressor. (b) Chirped pulse. (c) Residual group delay and tunable range of Dazzler (shadow). (d) Compressed pulse (black) and FTL pulse (red).

For a near-single-cycle 100 PW laser, only considering the on-axis pulse is insufficient. Using the model and method given in Supplementary Ref. [6], Supplementary Fig. 5(a) gives the simulated 3D spatiotemporal structure of the compressed pulsed beam when the FTL pulse is achieved using Dazzler. When this pulsed beam is focused by a 1 m focal length parabola, Supplementary Fig. 5(b) shows the

simulated 3D spatiotemporal structure of the focused pulsed beam, and the focused peak intensity would reach ~4×10^{23} W cm^{-2}.

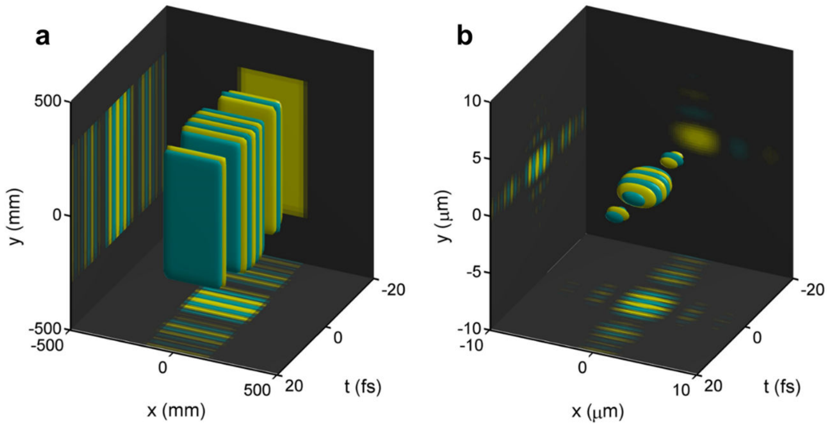

Supplementary Fig. 5 Supported pulsed beams with perfect gratings. (a) Compressed and (b) focused pulsed beams. The grating diffraction in the compressor happens in the x-z plane, and the focal length of the parabola is 1 m.

The above simulation results were performed assuming that the compressor is considered to deploy four perfect gratings with uniform diffraction efficiency maps and flat diffraction wavefronts. However, large-aperture perfect gratings cannot be achieved in an engineering implementation. In order to investigate the actual case, the effects of grating diffraction efficiency maps and grating diffraction wavefronts will be simulated in Supplementary Notes 3 and 4, respectively.

Supplementary Note 2:

Feasibility analysis of large-aperture ultra-broadband grating. Supplementary Fig. 6 illustrates the fabrication tolerance of the 1443 lines/mm ultra-broadband grating and the manufacturing accuracy of our team's meter-scale grating. The initial design grating structure is a line density of 1443 lines/mm, duty cycle of 0.634, shape factor of 1.91, and depth of 225 nm. The manufacturing accuracy is indicated by the yellow rectangle, where the accuracy of line density, duty cycle, shape factor, and depth are ± 0.5 lines/mm, ± 0.02, ± 0.3, and ± 3%. Obviously, the ultra-broadband grating is compatible with today's available grating manufacturing.

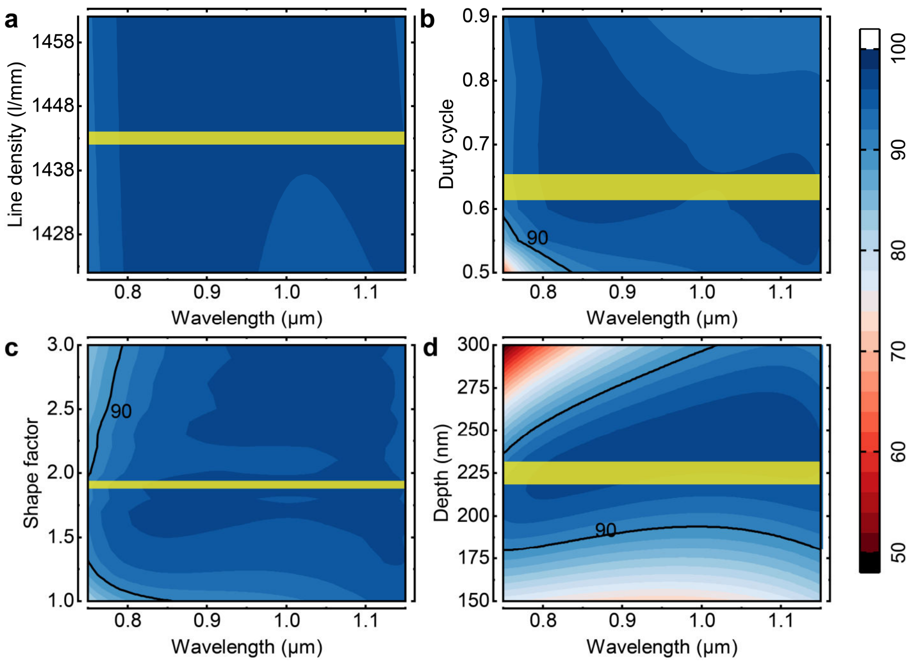

Supplementary Fig. 6 Fabrication tolerance of (a) line density, (b) duty cycle, (c) shape factor, and (d) depth of the 1443 lines/mm ultra-broadband grating. The yellow rectangle indicates the manufacturing accuracy of the current meter-scale grating.

Typical damage morphology. Supplementary Fig. 7 shows the damage morphologies of the ultra-broadband grating tested at a 15 fs laser pulse with a bandwidth over 200 nm near the center wavelength of 925 nm.

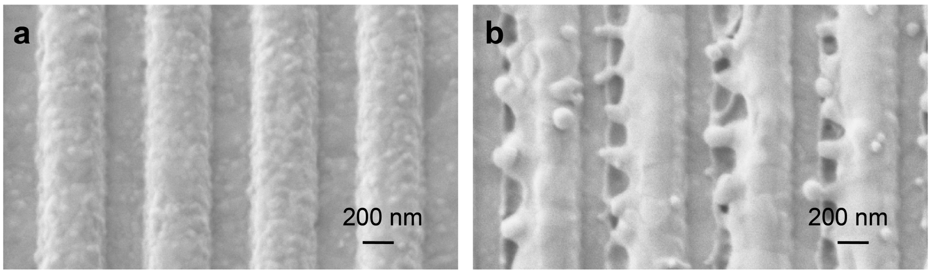

Supplementary Fig. 7 SEM images of the grating surface. (a) Undamaged grating. (b) Typical damage morphology. The ultra-broadband grating is tested at 50° with the illumination fluence >500 mJ cm^{-2}.

At the fluence above the LIDT, the irradiated corner of the grating ridge was ablated. Although only one image is presented here, this damage morphology appears in all the laser irradiation conditions. The above damage morphology is similar to that of the previously reported damage of gold gratings[7], and the LIDT shows no signs of degradation.

Supplementary Note 3:

Diffraction efficiency map vs. Compressed and focused pulse. Owing to the limited size of our original exposure system, the tiled meter-size grating was accepted. Supplementary Fig. 8 shows the measured diffraction efficiency map of a 970 mm × 370 mm grating in our group[8].The efficiency map is extremely uniform over the central 84% of the grating area, exhibiting an average efficiency of 86% with a standard deviation of 0.103%. As stated in Supplementary Note 2, the aperture of ultra-broadband gratings can be scaled to meter size or even larger in engineering. Consequently, to investigate the effect of the diffraction efficiency map of the ultra-broadband grating on the designed near-single-cycle 100 PW laser, Supplementary Fig. 8 can be directly scaled from 970 mm × 370 mm to 870 mm × 600 mm and 1370 mm × 600 mm, respectively. Moreover, the pulsed beams before and after focusing were re-simulated.

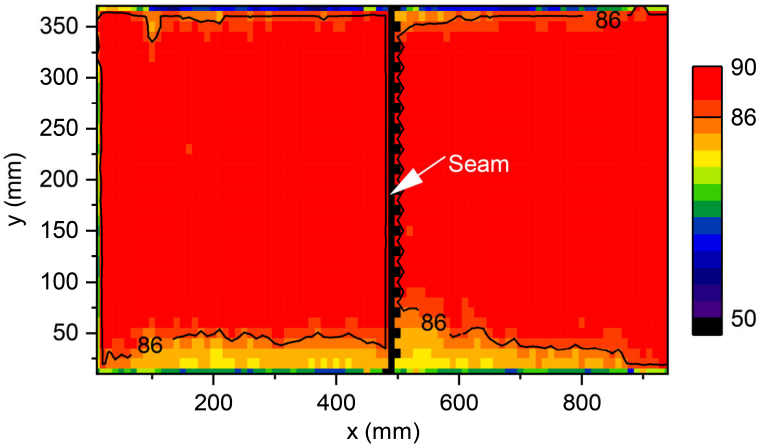

Supplementary Fig. 8 Scanning photometric diffraction efficiency map of a 970 mm× 370 mm at 750 nm. The blue area displays low diffraction efficiency owing to the seam.

Supplementary Fig. 9 shows the effects of the weak non-uniformity and seam of the diffraction efficiency map (see Supplementary Fig. 8) on the 3D spatiotemporal structure of the compressed and focused pulse beam. The following simulations can be divided into two main categories according to whether or not to consider the existence of seams in ultra-broadband gratings.

If seams are not considered (we have now built an upgraded meter-sized exposure system), Supplementary Fig. 9 (a) and (b) shows the compressed and focused pulse beams when diffraction efficiency maps are added to the four gratings G1-G4. The simulated 3D spatiotemporal structures have no significant changes compared to Supplementary Fig.5 (a) and (b).

Conversely, Supplementary Fig. 9 (c), (e), and (g) simulate the compressed pulse beams in the presence of a seam in Supplementary Fig. 8. When the non-uniform diffraction efficiency map is loaded on G1 and G4, a gap in the x-y plane appears in the compressed pulse beam [see Supplementary Fig. 9(c)]. In addition, the compressed pulsed beam exhibits spatiotemporal modulation in the x-t plane [see Supplementary Fig. 9 (e)] when G2 and G3 with the non-uniform diffraction efficiency maps. Accordingly, the compressed pulsed beam has a gap in the x-y plane and spatiotemporal modulation in the x-t plane [see Supplementary Fig. 9 (g)], where G1-G4 all loaded the diffraction efficiency map with seams.

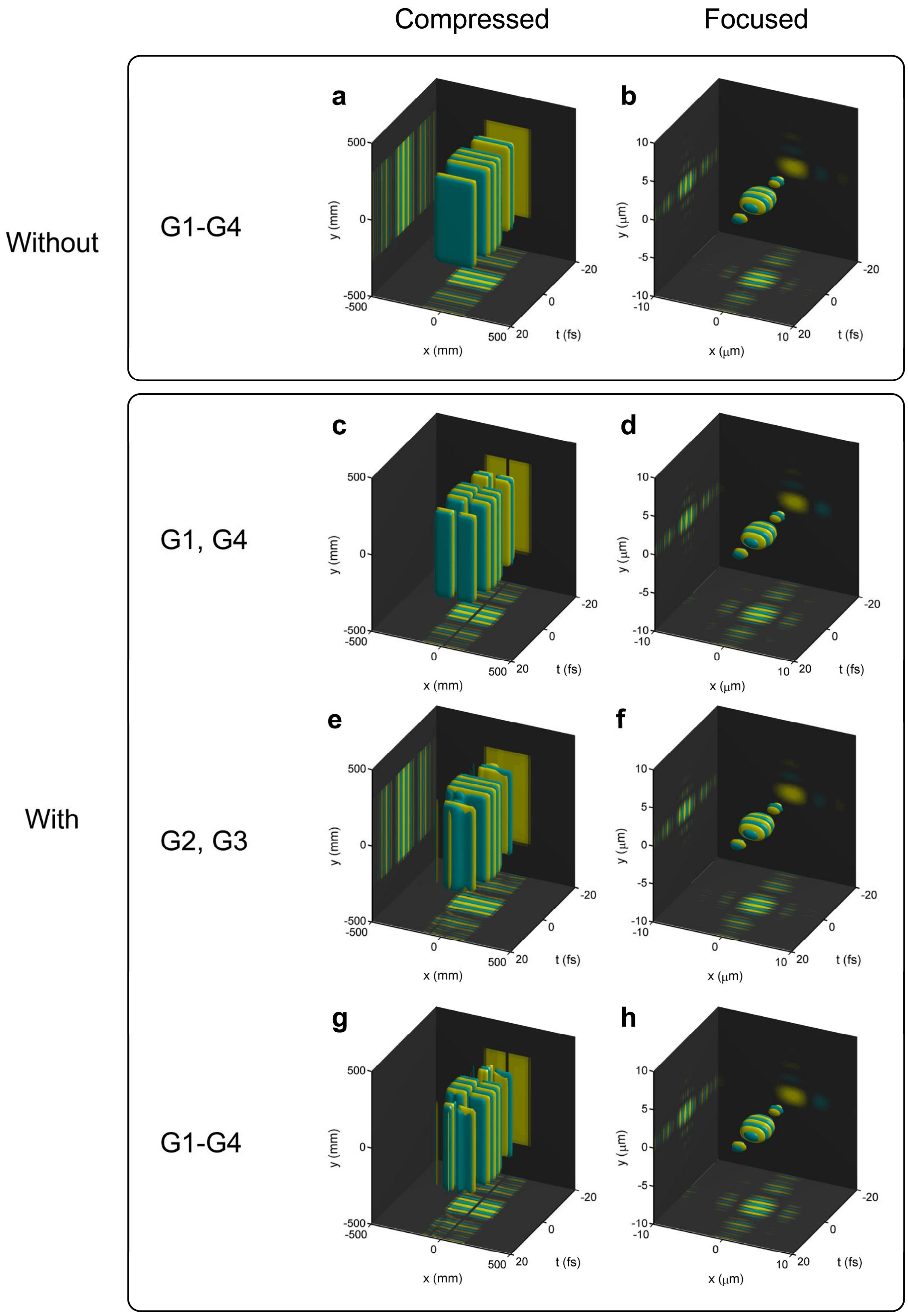

Supplementary Fig. 9 Supported pulsed beams with imperfect gratings. (a, c, e, g) Compressed and (b, d, f, h) focused pulsed beams. The ultra-broadband gratings (a-b) G1-G4 in the compressor were considered to load the diffraction efficiency map without seams in Supplementary Fig. 8. The ultra-broadband gratings (c-d) G1 and G4, (e-f) G2 and G3, and (g-h) G1-G4 in the compressor were considered to load the diffraction efficiency map with seams in Supplementary Fig. 8.

However, owing to the narrow width of the seam, the 3D spatiotemporal structure of the focused pulsed beam [see Supplementary Fig. 9 (d), (f), and (h)] almost keeps unchanged compared to

Supplementary Fig.5 (b).

Nevertheless, the focused peak intensity is different in the above cases. Here, the focused peak intensity in Supplementary Fig. 5 (b) (with perfect gratings) is normalized to 1. Accordingly, Supplementary Fig. 10 shows the focused peak intensities in Supplementary Figs. 9 (b), (d), (f), and (h) are 0.96, 0.85, 0.87, and 0.75, respectively. The seam in tiled gratings can reduce the focused peak intensity. Typically, large gratings are required at the second and the third gratings G2 and G3 (see Supplementary Fig. 1), and then the normalized degradation would be around 1 - 0.87 = 0.13. However, with a sufficiently large exposure system, the normalized degradation induced by the diffraction efficiency map (with no seam) is only around 1 - 0.96 = 0.04.

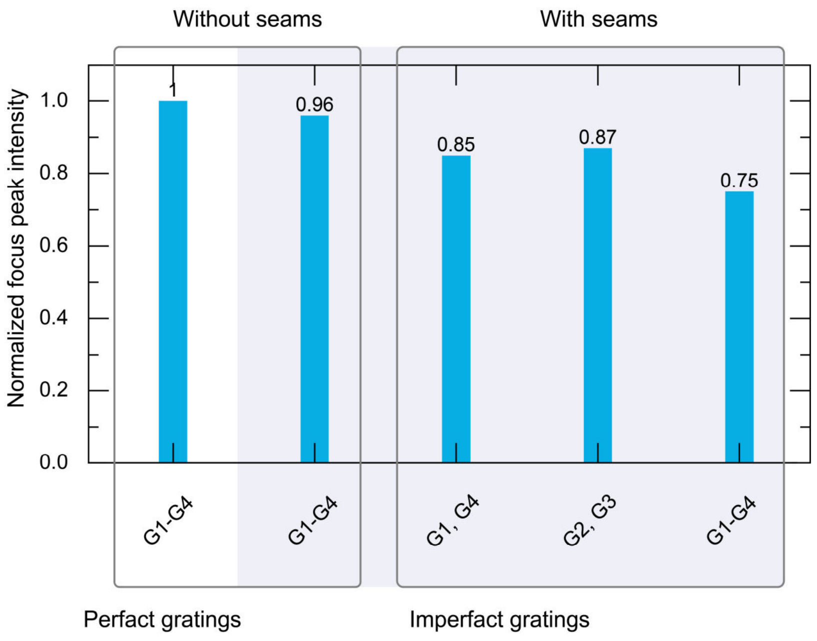

Supplementary Fig. 10 Degradation of focused peak intensity. The focused peak intensity with perfect gratings (i.e., uniform diffraction efficiency) is normalized to 1. Results of Supplementary Figs. 9 (b), (d), (f), and (h) are 0.96, 0.85, 0.87, and 0.75, respectively.

Supplementary note 4:

Diffraction wavefront vs. Compressed and focused pulse. Supplementary Fig. 11 shows the measured diffraction wavefront of a 970 mm×370 mm tiled grating. The wavefronts of the left and right parts that make up the tiled grating were measured separately. The measurement wavelength λ is 632.8 nm. The PV values of the left and the right gratings are approximately $\lambda/3$ and $\lambda/2$. In our current manufacturing technology, the wavefront presents a modulation of concentric rings. As stated in Supplementary Note 2, the aperture of ultra-broadband gratings can be scaled to meter size or even larger in engineering. Consequently, to investigate the effect of the diffraction wavefront of the ultra-broadband grating on the designed near-single-cycle 100 PW laser, the 3D spatiotemporal structures of compressed and focused pulsed beams were simulated directly using different concentric ring wavefronts in the following results.

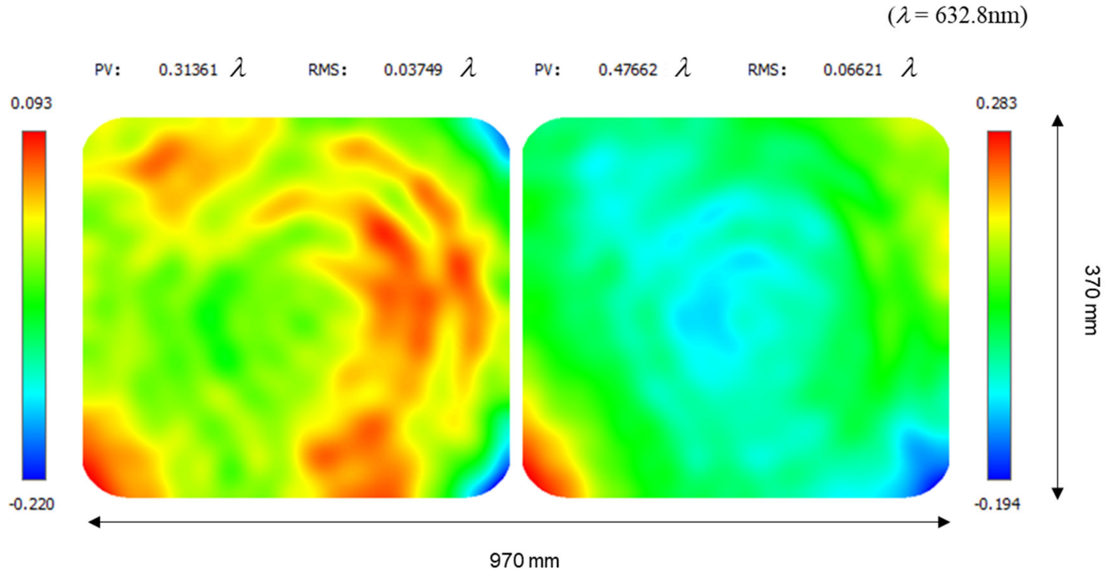

Supplementary Fig. 11 Measured diffraction wavefront of a 970 mm × 370 mm tiled grating. The PV values of the left and the right gratings are around $\lambda/3$ and $\lambda/2$. The measurement wavelength λ is 632.8 nm.

Since the wavefronts of G1 and G4 introduce only wavelength-independent distortion[6,9], which can be easily eliminated by the deformable mirror, only the wavefronts of G2 and G3 in the compressor are considered in the following analysis.

Supplementary Figs. 12 (a-f) show the 3D spatiotemporal structure of the compressed and focused pulse beams at a PV value of $\lambda/3$. When the modulation period of the wavefront is 800 mm [see Supplementary Fig. 12 (a)], both the compressed and the focused pulsed beams generate spatial distortions in the y-t plane and spatiotemporal distortions in the x-t plane [see Supplementary Fig. 12 (b) and (c)]. In contrast, when the modulation period of the wavefront is 200 mm [see Supplementary Fig. 12 (d)], distortions become more severe for both the compressed and the focused pulsed beams [see Supplementary Fig. 12 (e) and (f)] compared to the results in Supplementary Fig. 12 (b) and (c).

Similarly, Supplementary Figs. 12 (g-l) display the compressed and focused pulse beams at a PV value of $\lambda/2$. When the modulation period of the wavefront is 800 mm [see Supplementary Fig. 12 (g)],

distortions [see Supplementary Fig. 12 (h) and (i)] are slightly deteriorated compared to the results in Supplementary Fig. 12 (b) and (c). And, compared to the results in Supplementary Fig. 12 (e) and (f), distortions in Supplementary Fig. 12 (k) and (l) also deteriorate with a 200 mm modulation period [see Supplementary Fig. 12 (j)].

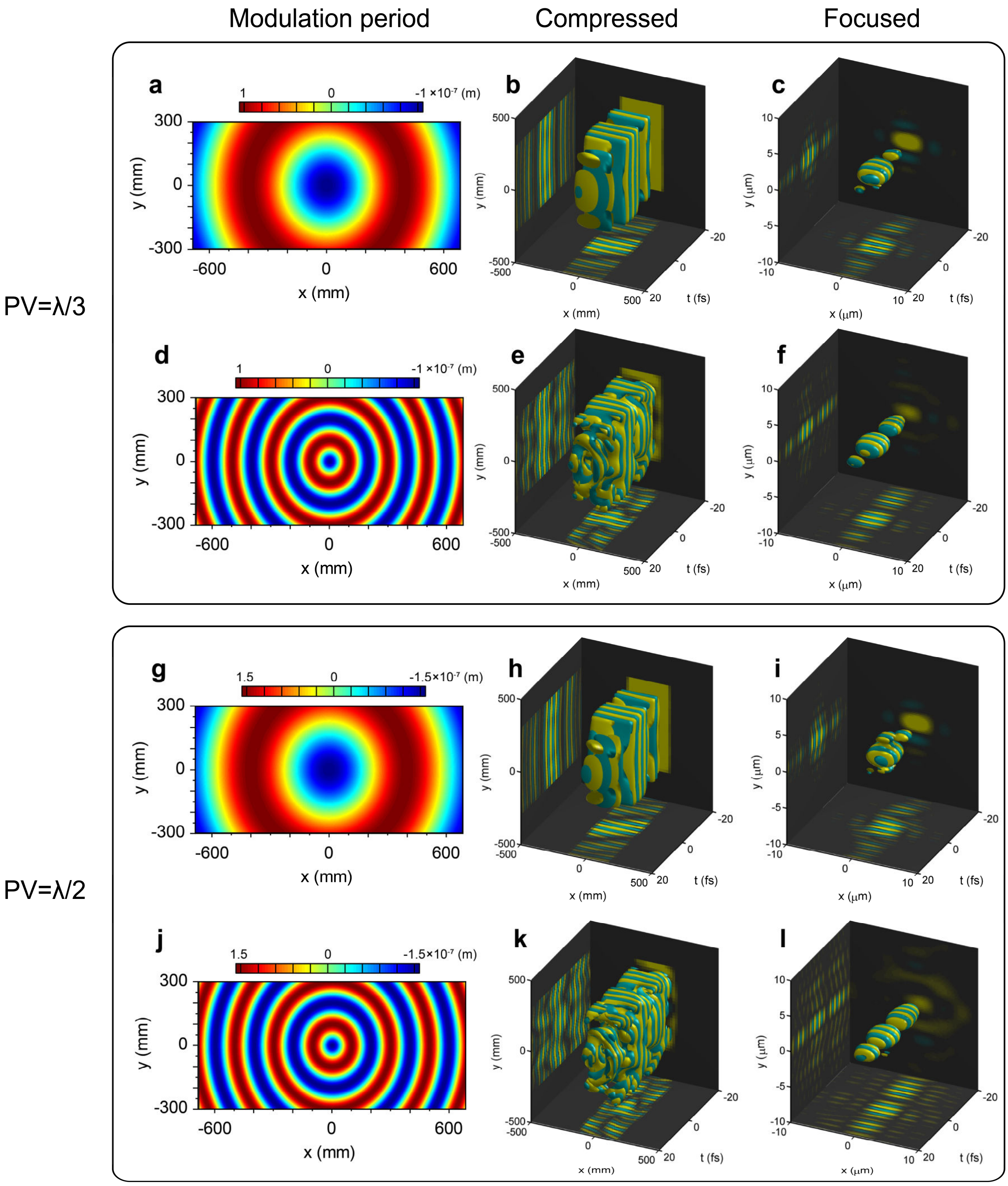

Supplementary Fig. 12 Supported pulsed beams with imperfect gratings. (a, d, g, j) Produced diffraction wavefront, (b, e, h, k) compressed and (c, f, i, l) focused pulsed beams. (a, g) The concentric ring wavefront has an 800 mm modulation period and (d, j) 200 mm modulation period. The concentric ring wavefront has (a-f) a $\lambda/3$ PV and (g-l) a $\lambda/2$ PV. The measurement wavelength λ is 632.8nm.

Supplementary Fig. 13 illustrates the values of the normalized focus peak intensity in the above cases. Here, the focus peak intensity in Supplementary Fig. 5 (with perfect grating) is normalized to 1.

The normalized peak focus intensity decreases from 0.71 to 0.60 and from 0.66 to 0.28 as the modulation period decreases from 800 mm to 200 mm at a PV value of $\lambda/3$ and $\lambda/2$, respectively. For the degradation of the normalized focus peak intensity to be less than 50%, the modulation period should be larger than 200 mm, and the modulation PV should be less than $\lambda/2$, which can be satisfied by our current grating manufacturing capability. In addition, this kind of wavefront distortions can now be controlled or pre-compensated[9, 10], and the method is shown in Supplementary Fig. 14 and introduced in Refs. [9, 10].

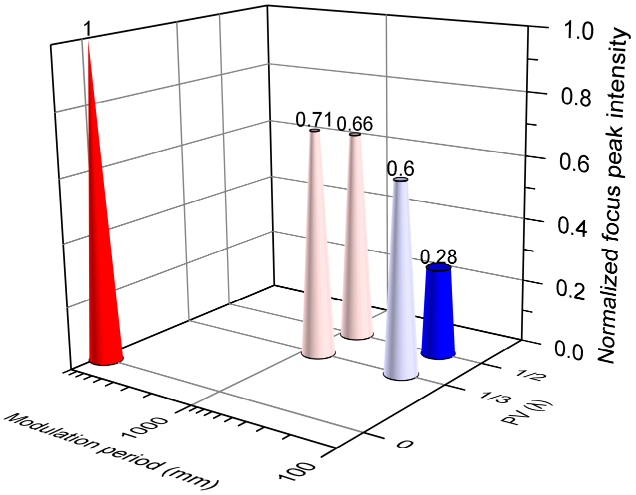

Supplementary Fig. 13 Degradation of focus peak intensity. The focused peak intensity with perfect gratings (i.e., flat wavefront L = ∞, PV = 0) is normalized to 1. Results of Supplementary Figs. 12 (c) (L = 800 mm, PV = 1/3 λ), Supplementary Figs. 12 (f) (L = 200 mm, PV = 1/3 λ), Figs. S12 (i) (L = 800 mm, PV = 1/2 λ) and Supplementary Figs. 12 (l) (L = 200 mm, PV = 1/2 λ) are 0.71, 0.60, 0.66, and 0.28, respectively. L, modulation period; λ = 632.8 nm.

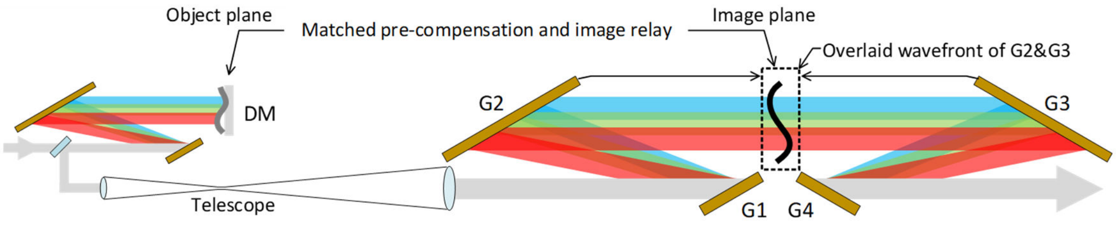

Supplementary Fig. 14 Pre-compensation of grating wavefronts in the compressor. Reproduced with permission.[10] Copyright 2022, Wiley. Wavelength-dependent wavefront correction is introduced and imaged from a small compressor into the main compressor.

Supplementary References

1. Li, Z., Kato, Y. & Kawanaka, J. Simulating an ultra-broadband concept for Exawatt-class lasers. *Sci. Rep.* **11**, 1–16 (2021).

2. Mourou, G., Mironov, S., Khazanov, E. & Sergeev, A. Single cycle thin film compressor opening the door to Zeptosecond-Exawatt physics. *Eur. Phys. J. Spec. Top.* **223**, 1181–1188 (2014).

3. Kafka, K. R. P. *et al.* Few-cycle pulse laser induced damage threshold determination of ultra-broadband optics. *Opt. Express* **24**, 28858–28868 (2016).

4. Poole, P., Trendafilov, S., Shvets, G., Smith, D. & Chowdhury, E. Femtosecond laser damage threshold of pulse compression gratings for petawatt scale laser systems. *Opt. Express* **21**, 26341–26351 (2013).

5. Dazzler UHR-650-1100. 6600 https://fastlite.com/wp-content/uploads/spec-uhr-650-1100-rev2018-1.pdf (2018).

6. Li, Z., Liu, J., Xu, Y., Leng, Y. & Li, R. Simulating spatiotemporal dynamics of ultra-intense ultrashort lasers through imperfect grating compressors. *Opt. Express* **30**, 41296-41312 (2022).

7. Han. Y., Jin. Y., Kong. F., Wang. Y., Zhang. Y., Cao. H., Cui. Y. & Shao. J. High-repetition-rate and multi-pulse ultrashort laser damage of gold-coated photoresist grating. *Appl Surf Sci* **576**, 151819 (2022).

8. Z. Yu, W. Shenghao, L. Shijie, S. Jianda, J. Yunxia & X. Zhilin. Fast measurement technique for obtaining the diffraction efficiency and its uniformity of a large-aperture pulse compression grating. Proc.SPIE. 2019. p. 1083918.

9. Li, Z. & Kawanaka, J. Complex spatiotemporal coupling distortion pre-compensation with double-compressors for an ultra-intense femtosecond laser. *Opt. Express* **27**, 25172-25186 (2019).

10. Li, Z, Leng, Y. & Li, R. Further Development of the Short-Pulse Petawatt Laser: Trends, Technologies, and Bottlenecks. *Laser Photonics Rev.* **17**, 2100705 (2022).
